# Enrofloxacin and Toltrazuril Are Able to Reduce *Toxoplasma gondii* Growth in Human BeWo Trophoblastic Cells and Villous Explants from Human Third Trimester Pregnancy

**DOI:** 10.3389/fcimb.2017.00340

**Published:** 2017-07-26

**Authors:** Rafaela J. da Silva, Angelica O. Gomes, Priscila S. Franco, Ariane S. Pereira, Iliana C. B. Milian, Mayara Ribeiro, Paolo Fiorenzani, Maria C. dos Santos, José R. Mineo, Neide M. da Silva, Eloisa A. V. Ferro, Bellisa de Freitas Barbosa

**Affiliations:** ^1^Laboratory of Immunophysiology of Reproduction, Institute of Biomedical Science, Federal University of Uberlândia Uberlândia, Brazil; ^2^Departament of Morphology, Federal University of Triângulo Mineiro Uberaba, Brazil; ^3^Department of Medical, Surgery and Neuroscience, University of Siena Siena, Italy; ^4^Department of Gynecology and Obstetrics, Faculty of Medicine, Federal University of Uberlândia Uberlândia, Brazil; ^5^Laboratory of Immunoparasitology, Institute of Biomedical Science, Federal University of Uberlândia Uberlândia, Brazil; ^6^Laboratory of Immunopathology, Institute of Biomedical Science, Federal University of Uberlandia Uberlândia, Brazil

**Keywords:** *Toxoplasma gondii*, trophoblast, placenta, enrofloxacin, toltrazuril, treatment

## Abstract

Classical treatment for congenital toxoplasmosis is based on combination of sulfadiazine and pyrimethamine plus folinic acid. Due to teratogenic effects and bone marrow suppression caused by pyrimethamine, the establishment of new therapeutic strategies is indispensable to minimize the side effects and improve the control of infection. Previous studies demonstrated that enrofloxacin and toltrazuril reduced the incidence of *Neospora caninum* and *Toxoplasma gondii* infection. The aim of the present study was to evaluate the efficacy of enrofloxacin and toltrazuril in the control of *T. gondii* infection in human trophoblast cells (BeWo line) and in human villous explants from the third trimester. BeWo cells and villous were treated with several concentrations of enrofloxacin, toltrazuril, sulfadiazine, pyrimethamine, or combination of sulfadiazine+pyrimethamine, and the cellular or tissue viability was verified. Next, BeWo cells were infected by *T. gondii* (2F1 clone or the ME49 strain), whereas villous samples were only infected by the 2F1 clone. Then, infected cells and villous were treated with all antibiotics and the *T. gondii* intracellular proliferation as well as the cytokine production were analyzed. Finally, we evaluated the direct effect of enrofloxacin and toltrazuril in tachyzoites to verify possible changes in parasite structure. Enrofloxacin and toltrazuril did not decrease the viability of cells and villous in lower concentrations. Both drugs were able to significantly reduce the parasite intracellular proliferation in BeWo cells and villous explants when compared to untreated conditions. Regardless of the *T. gondii* strain, BeWo cells infected and treated with enrofloxacin or toltrazuril induced high levels of IL-6 and MIF. In villous explants, enrofloxacin induced high MIF production. Finally, the drugs increased the number of unviable parasites and triggered damage to tachyzoite structure. Taken together, it can be concluded that enrofloxacin and toltrazuril are able to control *T. gondii* infection in BeWo cells and villous explants, probably by a direct action on the host cells and parasites, which leads to modifications of cytokine release and tachyzoite structure.

## Introduction

*Toxoplasma gondii* is an obligate intracellular protozoan parasite able to infect many cell types in warm-blooded vertebrates (Buxton et al., 2007). It is estimated that one third of the population in the world is infected by this parasite, making it one of the most successful parasites (Montoya and Liesenfeld, 2004). In immunocompetent hosts, the toxoplasmosis is generally asymptomatic (Montoya and Liesenfeld, 2004). However, if maternal infection by *T. gondii* occurs during pregnancy, the embryo or fetus is at risk of developing congenital toxoplasmosis, due to transplacental transmission of the parasite (Kodjikian, 2010). The primary infection during pregnancy can result in miscarriage, stillbirth, premature birth, malformations, and neurological and/or ocular disorders in newborns (Carlier et al., 2012; Li et al., 2014; Oz, 2014). Thus, congenital toxoplasmosis is a severe public health problem in many countries, including Brazil (Dubey et al., 2012; Carellos et al., 2014).

A Th1-type immune response against *T. gondii* is observed during infection, with the participation of pro-inflammatory cytokines as interferon (IFN)-γ and interleukin (IL)-12 (Filisetti and Candolfi, 2004). During infection, macrophages, neutrophils, and dendritic cells produce IL-12, which activates CD4^+^ T lymphocytes to produce IFN-γ, triggering several anti-parasitic mechanisms in macrophages and natural killer cells (Gazzinelli et al., 1994; Lang et al., 2007; Denkers, 2010). Additionally, other pro-inflammatory cytokines play an important role in *T. gondii* infection, as macrophage migration inhibitory factor (MIF), tumor necrosis factor (TNF) and IL-6 (Filisetti and Candolfi, 2004; Lang et al., 2007; Flores et al., 2008; Mirpuri and Yarovinsky, 2012; Castro et al., 2013; Tomar and Singh, 2014). Thus, the production of these pro-inflammatory cytokines represents a solid and classical mechanism of immunological defense associated with the control of *T. gondii* infection in the host. However, to regulate this pro-inflammatory profile, anti-inflammatory cytokines as IL-10 and transforming growth factor (TGF)-β are necessary to avoid an exacerbated immune response, which could be harmful to the host (Filisetti and Candolfi, 2004). Although, the hosts infected by *T. gondii* activate this immunological response, it is not sufficient to clear the infection. In this sense, the use of drugs to control the infection is mandatory, especially in infected pregnant and congenitally infected children.

Currently, there are few drugs available for the treatment of congenital toxoplasmosis. If there is no evidence of fetal transmission, spiramycin is used to prevent vertical transmission (Peyron et al., 2017). This drug is a macrolide antibiotic that does not cross the placenta (Montoya and Remington, 2008). When fetal infection is confirmed, the first choice of treatment is the combination of pyrimethamine plus sulfadiazine. These drugs act in synergism on *T. gondii* folate synthesis by the inhibition of dihydropteroate synthase (DHPS) and dihydrofolate reductase (DHFR), two important enzymes for parasite survival and replication (Villena et al., 1998; Doliwa et al., 2013). The co-administration of folinic acid is necessary to minimize the toxic effects of pyrimethamine; due to the bone marrow suppression in mothers and newborns, and due to its teratogenic effects. Then, it is necessary to avoid using this drug during the first trimester of pregnancy (Montoya and Liesenfeld, 2004; Oz, 2014). Moreover, sulfadiazine is associated with gastrointestinal disorders, and patients often do not tolerate this chemotherapy (Montoya and Liesenfeld, 2004). Additionally, more than half of patients treated with spiramycin retained *Toxoplasma* DNA in their blood or remained infected (Habib, 2008). Thus, to find active and less toxic drugs as new therapeutic strategies to prevent or treat toxoplasmosis congenital is mandatory.

Additional alternative drugs have shown useful effects against *T. gondii in vitro*. Azasterols, developed as inhibitors of sterol biosynthesis, demonstrated an ability to inhibit *T. gondii* proliferation by acting against the mitochondria of the parasite (Dantas-Leite et al., 2004; Martins-Duarte et al., 2011). Also, *Artemisia annua* infusion treatment was able to decrease the mortality of C57BL/6 mice infected with *T. gondii* RH or ME49 strains (de Oliveira et al., 2009) and reduced the vertical transmission of the *T. gondii* ME49 strain in *Calomys callosus* rodents (Costa et al., 2009). Finally, previous studies have demonstrated the effect of enrofloxacin against protozoan parasites, including *T. gondii* and *Neospora caninum* (Gottstein et al., 2005; Delespaux et al., 2010; Barbosa et al., 2012).

Enrofloxacin is a fluoroquinolone antibiotic class with broad spectrum and is commonly used in veterinary medicine, mainly for treatments of respiratory and intestinal infections (Devreese et al., 2014). It acts against Enterobacteriaceae, Gram negative bacteria, as *Pseudomonas aeruginosa*, as well as in some Gram-positive bacteria (Martinez et al., 2006). Fluoroquinolones are known to inhibit DNA replication from bacteria, since the target is the DNA gyrase and topoisomerase IV (Collin et al., 2011; Martins-Duarte et al., 2015). Moreover, many studies demonstrated the effect of enrofloxacin against infections caused by parasite protozoans, although the target of this drug in these protozoans is still unknown (Gottstein et al., 2005; Barbosa et al., 2012). Enrofloxacin was able to control the infection by *N. caninum* in the brain of pregnant females from the C57/BL6 lineage (Gottstein et al., 2005). In addition, our previous study demonstrated that enrofloxacin was more efficient for the control of the invasion and replication of *T. gondii* in human fibroblast cells (HFF line) compared to sulfadiazine and pyrimethamine (Barbosa et al., 2012). Furthermore, enrofloxacin was able to reduce the number of parasites and immunopathology in the brain of *C. callosus* infected by *T. gondii* (Barbosa et al., 2012).

Toltrazuril is a drug that is mostly used in veterinary medicine to treat, control or prevent different coccidiosis in birds, pigs, cattle, sheep, and goats (Olsen et al., 2012). Toltrazuril is derived from a triazone and this drug was shown to be efficient for the control of *T. gondii* infection, acting on several forms of the parasite, such as sexual or asexual stages in the cat gut (Haberkorn et al., 1996; Kul et al., 2013). Ponazuril is the active principle, which induces mitochondrial damage and inhibits cellular division of the parasite (Mitchell et al., 2004). Previous *in vivo* and *in vitro* studies have demonstrated that toltrazuril is effective against other protozoa as *Eimeria* sp., *Isospora* sp., and *N. caninum* (Haberkorn et al., 2001; Kul et al., 2013). Additionally, toltrazuril completely reduced the number of *Eimeria* sp. oocysts in young goats (Iqbal et al., 2013). Also, toltrazuril has been reported to be a good strategy for the treatment of congenital neosporosis in mice (Gottstein et al., 2005).

Our group has investigated several aspects of immumodulation mechanisms at the maternal-interface using BeWo cells as a trophoblast model, including their cross-talk with monocytes (Castro et al., 2013) and the role of cytokines and drugs during *T. gondii* infection (Franco et al., 2011; Barbosa et al., 2014, 2015). Even though BeWo cells are derived from a human choriocarcinoma, they are becoming an excellent *in vitro* model to investigate *T. gondii* infection in trophoblast cells (Franco et al., 2011; Castro et al., 2013; Barbosa et al., 2014, 2015), since they conserve many characteristics of the normal human trophoblast cells, such as the trophoblast/epithelial marker CK7 (Abou-Kheir et al., 2017), and cytokine and hormone production (Pattillo and Gey, 1968). In addition, our group has been investigating the *T. gondii* infection in human villous explants from the first and third trimester as an experimental model of the maternal-fetal interface (de Oliveira Gomes et al., 2011; Castro-Filice et al., 2014). Therefore, studies about new therapeutic strategies against *T. gondii* vertical transmission should be performed in models of trophoblast cells and the maternal-fetal interface in order to determine alternative drugs to improve the prevention or treatment of congenital toxoplasmosis.

Our previous studies demonstrated that BeWo cells and villous explants infected by *T. gondii* release MIF and IL-6 (de Oliveira Gomes et al., 2011; Franco et al., 2011; Barbosa et al., 2015). Furthermore, we previously showed that BeWo cells treated with MIF or IL-6 recombinant cytokines significantly reduced the *T. gondii* proliferation, evidencing the important role of these mediators in the control of infection during a pregnancy (Barbosa et al., 2014, 2015). Then, the upregulation of these cytokines can be a mechanism triggered by the host cells, as trophoblast cells, to control the *T. gondii* infection.

Thus, considering the beneficial effects of enrofloxacin and toltrazuril against *T. gondii* infection and to other parasites from the Apicomplexa phylum, the aim of the present study was to evaluate the role of these drugs in the susceptibility of BeWo trophoblastic cells and human villous explants to *T. gondii*.

## Materials and methods

### BeWo cell culture

Human trophoblast cells (BeWo line) were acquired from American Type Culture Collection (ATCC, Manassas, VA, USA) and maintained in culture flasks of 25 or 75 cm^2^ in RPMI 1640 medium (Cultilab, Campinas, SP, Brazil) supplemented with 100 U/mL of penicillin (Sigma Chemical Co., St. Louis, MO, USA), 100 μg/mL of streptomycin (Sigma) and 10% fetal bovine serum (FBS) (Cultilab) in a humidified incubator at 37°C and 5% CO_2_ (Barbosa et al., 2008). The Ethics Committee of the Federal University of Uberlândia, MG, Brazil, communicates that studies performed with cell lines acquired commercially do not need ethical approval (Protocol # 13/2012).

### Human placenta samples and chorionic villous explants cultures

Third-trimester human placentas (*n* = 6) were acquired from pregnant patients after elective cesarean section deliveries (36–40 weeks of pregnancy) at the Clinics Hospital of the Federal University of Uberlândia (HC-UFU), MG, Brazil. Placental tissues were collected only if the patients did not show evidence of pre-eclampsia, hypertension, cardiac disease, diabetes, infectious diseases such as toxoplasmosis, and other manifestations which could interfere with the results of this study. The collection of the placental tissue was authorized in accordance with Ethics Committee of the Federal University of Uberlândia, MG, Brazil (Approval Number: 1.585.342).

After being collected, the placental tissue was washed with sterile phosphate-buffered saline (PBS) in order to remove any excess blood, and the dissection of the villous was performed using a stereomicroscope to eliminate endometrial tissue and fetal membranes up to 1 h after collection. Then, floating terminal chorionic villi were collected, placed in 96-well plates (one per well) and cultured in 200 μL RPMI 1640 medium supplemented with 10% FBS, penicillin and streptomycin for 24 h at 37°C and 5% CO_2_ for future experiments. The volume of the villous explants was ~10 mm^3^ (de Oliveira Gomes et al., 2011; Castro-Filice et al., 2014).

### Parasites

Tachyzoites from *T. gondii* (2F1 clone), derived from the highly virulent RH strain and expressing the β-galactosidase gene, were kindly provided from Dr. Vern Carruthers, Medicine School of Michigan University (USA). In parallel, *T. gondii* tachyzoites from ME49 strain (moderate virulence) were provided from Dr. Karine Resende, Federal University of Triângulo Mineiro, MG, Brazil. Both parasite strains were maintained in culture flasks containing BeWo cells in RPMI 1640 medium supplemented with penicillin, streptomycin and 2% FBS at 37°C and 5% CO_2_ (Angeloni et al., 2013; Barbosa et al., 2015).

### Treatment of BeWo cells and human chorionic villous explants with drugs and the viability assay

In the first step of experiments, it was verified whether the drugs selected for treatment protocols could be toxic for BeWo cells and to determine the best concentration of each drug for further experiments. The tetrazolium salt colorimetric (MTT) assay was performed, as previously described by Mosmann (1983).

For this purpose, BeWo cells were cultured in 96-well plates (3 × 10^4^ cells/well/200 μL) for 24 h in RPMI 1640 medium with 10% FBS at 37°C and 5% CO_2_. Next, the cells were treated with sulfadiazine (Sigma), enrofloxacin (Bayer Healthcare, São Paulo, SP, Brazil), or toltrazuril (Bayer Healthcare) for an additional 24 h in RPMI 1640 medium containing 5% FBS as follows: 1.56, 3.125, 6.25, 12.5, 25, 50, 100, or 200 μg/mL (Barbosa et al., 2012). At the same time, BeWo cells were also treated with pyrimethamine (Sigma) for 24 h, as follows: 0.0624, 0.125, 0.250, 0.500, 1, 2, 4, 8, 12, 16, or 20 μg/mL in RPMI 1640 medium with 5% FBS. Finally, the combination of sulfadiazine and pyrimethamine was used also for 24 h, as follows: 1.56 + 0.0624, 3.125 + 0.125, 6.25 + 0.250, 12.5 + 0.500, 25 + 1, 50 + 2, 100 + 4, or 200 + 8 μg/ml, respectively. The different concentrations of pyrimethamine were based on the proportion corresponding to 4% of the selected doses for sulfadiazine (Derouin and Chastang, 1989; Meneceur et al., 2008; Jin et al., 2009). Enrofloxacin and toltrazuril were diluted in RPMI medium only. However, sulfadiazine and pyrimethamine were dissolved in RPMI 1640 medium containing dimethyl sulfoxide (DMSO) in order to improve the dilution. To verify whether DMSO could be toxic to BeWo cells, we treated the cells with 0.26% of DMSO in RPMI 1640, the percentage used in the treatments with pyrimethamine, sulfadiazine, or combination of sulfadiazine plus pyrimethamine. Sulfadiazine or/and pyrimethamine were used as classical drugs in order to compare them with the alternative drugs proposed here, while the negative control was BeWo cells treated with RPMI 1640 medium containing 5% FBS only.

After these treatment protocols, the supernatants were removed and the cells were treated with 10 μL of MTT plus 90 μL medium with 10% FBS for 4 h in the same culture condition. The formazan crystals were solubilized by adding a solution containing 10% sodium dodecyl sulfate (SDS, Sigma) and 50% *N*,*N*-dimethyl formamide (Sigma) for 30 min (Mosmann, 1983). The optical densities were measured at 570 nm in a plate reader (Titertek Multiskan Plus, Flow Laboratories, McLean, VA, USA). Data were expressed as the percentage of viable cells (cellular viability %) in comparison to untreated cells (100% of cellular viability). Three independent experiments with nine replicates were performed for each condition.

In the second step of experiments, the villous explants were collected as described above and cultured for 24 h in RPMI 1640 medium with 10% FBS at 37°C and 5% CO_2_ to analyze the toxicity of the different concentrations of the drugs used in this study. The concentrations used were 600, 700, or 800 μg/mL for enrofloxacin; and 500, 800, or 900 μg/mL for toltrazuril. The combination of sulfadiazine and pyrimethamine (150 + 200 μg/mL, respectively; Castro-Filice et al., 2014) was used for comparison with the alternative drugs proposed here. The villi were treated with the different concentrations drugs for 24 h in RPMI 1640 medium containing 10% FBS. As negative controls, the villi were cultured with 10% FBS medium only, corresponding to 100% viability. After 24 h of treatments, the supernatants were collected for posterior measurements of lactate dehydrogenase (LDH) levels as the expression of drug toxicities (Castro-Filice et al., 2014), according to the manufacturer's instructions (LDH Liquiform, Labtes Diagnostica S.A., Lagoa Santa, MG, Brazil). This method is based on consumption and reduction of the absorption of NADH at 340 nm, which is measured in a DU-70 spectrophotometer (Beckman, Brea, CA., USA) for 2 min at 37°C. Data were shown in U/L of LDH enzymatic activity. In parallel, in order to verify the integrity of villous explants after treatments, they were collected for morphological analyses by hematoxylin and eosin staining. Three placentas were used, and three independent experiments were performed in five replicates.

### *T. gondii* infection (2F1 clone) and drug treatments in BeWo cells

Initially, we verified the *T. gondii* intracellular proliferation using a highly virulent strain (RH strain, 2F1 clone) in BeWo cells treated or not with different drugs. For this purpose, BeWo cells were cultured in 96-well plates (3 × 10^4^/200 μL/well) for 24 h in RPMI 1640 medium with 10% FBS at 37°C and 5% CO_2_. Next, cells were infected with *T. gondii* tachyzoites in the proportion of 5 parasites per cell (5:1) in medium containing 2% FBS. After 3 h, the medium was changed in order to remove extracellular parasites and the cells were treated as follows: (i) enrofloxacin (50 or 100 μg/mL); (ii) toltrazuril (25 or 50 μg/mL); (iii) sulfadiazine (100 or 200 μg/mL); (iv) pyrimethamine (4 or 8 μg/mL); or (v) combination of sulfadiazine and pyrimethamine (100 + 4 or 200 + 8 μg/mL, respectively). For negative controls, the conditions were as follows: uninfected cells and those treated with only medium (medium); or infected cells, but treated with only medium (*T. gondii*). In parallel, BeWo cells were cultured in 96-well plates (3 × 10^4^/200 μL/well) for 24 h in RPMI 1640 medium with 10% FBS, treated with the different drugs, but not infected. After 24 h of infection and/or treatment, the cell-free supernatants were collected and frozen at −80°C for later measurements of cytokines. In parallel, the *T. gondii* intracellular proliferation assay was performed in the infected cells by the β-galactosidase colorimetric reaction, as previously described (Castro et al., 2013; Barbosa et al., 2014, 2015). *T. gondii* intracellular proliferation (number of tachyzoites) was obtained according to a reference curve containing free tachyzoites (from 1 × 10^6^ to 15.6 × 10^3^). The data were expressed as a percentage (%) of *T. gondii* proliferation: the mean number of tachyzoites from controls (untreated and infected cells) corresponded to 100% parasite proliferation, then the number of tachyzoites from each treatment condition was transformed in percentage according to 100% of parasite proliferation from the control. Three independent experiments in nine replicates were performed.

### *T. gondii* infection (ME49 strain) and drugs treatment in BeWo cells

To determine the *T. gondii* intracellular proliferation using a moderate virulent strain (ME49), BeWo cells were cultured on 13 mm round glass slides (Sigma) in 24-well plates at a proportion of 1 × 10^5^ cells/200 μL medium with 10% FBS at 37°C and 5% CO_2_. After 24 h, the cells were infected at a ratio of 5:1 for 3 h. After, the cells were washed to remove extracellular parasites, and the cells were treated with the following drug concentrations: enrofloxacin, 100 μg/mL; toltrazuril, 12.5 μg/mL; sulfadiazine, 200 μg/mL; pyrimethamine, 8 μg/mL; or combination of sulfadiazine and pyrimethamine, 200 + 8 μg/mL. As controls, the cells were infected, but not treated (*T. gondii*), or uninfected and not treated (medium). After an additional 24 h, the supernatants were collected and stored at −80°C for the subsequent measurement of cytokines. The round glass slides with adherent cells were washed with sterile PBS, fixed with formaldehyde 10% for 24 h and stained with 1% toluidine blue for 10 s (Barbosa et al., 2008). Next, the cells were counted under a light microscope (BX40, Olympus, Tokyo, Japan) in order to determine the infection index (number of infected cell per 200 examined cells) and *T. gondii* intracellular proliferation (total number of tachyzoites per 200 examined cells; Barbosa et al., 2012). Three independent experiments of six replicates were performed.

### *T. gondii* infection (2F1 clone) and drugs treatment in human chorionic villous explants

In a third step of experiments, the *T. gondii* intracellular proliferation was investigated in villous explants treated or not with the panel of selected drugs. The villi were cultured in 96-well plates for 24 h in RPMI 1640 medium with 10% FBS at 37°C and 5% CO_2_. Next, the villi were infected with tachyzoites of the RH strain (2F1 clone) in a proportion of 1 × 10^6^ parasites to each well in RPMI 1640 medium with 10% FBS. After 24 h, the villi were washed with medium to remove the extracellular parasites and then treated for an additional 24 h with different concentrations of the drugs, as follows: (i) enrofloxacin (700 μg/mL); (ii) toltrazuril (900 μg/mL); or (iii) combination of sulfadiazine plus pyrimethamine (150 + 200 μg/mL), according to the results of the toxicity assay (LDH measurement) and morphological analyses. Villous explants, either uninfected and untreated (medium), or infected and untreated (*T. gondii*), were cultured with only medium as negative controls. In parallel, villous explants were treated with the different drugs for 24 h, but not infected. Next, the cell-free supernatants were collect and stored at −80°C for the later measurement of cytokines, while the villi were collected for parasite intracellular proliferation by the β-galactosidase colorimetric assay (Castro et al., 2013; Barbosa et al., 2014, 2015) or immunohistochemistry assay.

The parasite intracellular proliferation in villous explants samples was performed by adding 150 μL RIPA buffer [50 mM Tris-HCl, 150 mM NaCl, 1% Triton X-100, 1% (w/v) sodium deoxycholate, and 0.1% (w/v) sodium dodecyl sulfate (SDS), pH 7.5] supplemented with protease inhibitor cocktail (Complete, Roche Diagnostic, Mannheim, Germany) to each villous and homogenizing the samples in ice for protein extraction. The homogenate was centrifuged at 21,000 × *g* for 15 min at 4°C and the supernatant was collected to measure the protein total (μg/mL) using the Bradford assay (Bradford, 1976). Aliquots of 20 μL of each sample were used to determine *T. gondii* intracellular proliferation by β-galactosidase assay, as described above. Next, the data of number of tachyzoites were normalized according to the protein concentration of each villous, showing the number of tachyzoites per μg of tissue. Finally, the data were expressed as percentage (%) of *T. gondii* proliferation: the mean number of tachyzoites from controls (untreated and infected villous) corresponded to 100% of parasite proliferation, and the number of tachyzoites from each treatment condition was transformed into a percentage according to 100% of parasite proliferation from the control. Three samples of placenta were collected and three independent experiments with nine replicates were performed.

### Immunohistochemistry

To verify the immunolocalization of the parasites, villous explants were fixed in 10% buffered formalin, dehydrated in increasing alcohol concentrations, and embedded in paraffin. Sections with 4 μm were placed on glass slides and subjected to immunohistochemical analysis (de Oliveira Gomes et al., 2011; Castro-Filice et al., 2014). Briefly, for antigenic retrieval, sections were covered with citric acid pH 6.0 for 5 min in a microwave. To block endogenous phosphatase activity and reduce the non-specific binding, the sections were incubated with 5% acetic acid solution for 8 min at room temperature and 2.5% goat serum for 45 min at 37°C, respectively. Next, sections were incubated overnight at 4°C with *C. callosus* serum previously infected with *T. gondii* (1:100). On the following day, biotinylated goat-anti mouse IgG (1:600, Jackson Immuno Research Laboratories, West Grove, PA) secondary antibody was added to the section for 1 h at 37°C. The reaction was developed with fast red naphthol (Sigma), the tissue counterstained with Harris's hematoxylin and analyzed under a light microscope (BX40, Olympus, Tokyo, Japan; de Oliveira Gomes et al., 2011; Castro-Filice et al., 2014).

### Cytokines

Human cytokines IL-12p70, IL-6, TNF-α, MIF, IFN-γ, IL-10, and TGF-β1 were measured in supernatants of BeWo cells and villous explants by ELISA, according with manufacturer's instructions (R&D Systems, Minneapolis, MN, USA; or BD Biosciences, San Diego, CA, USA). Data were expressed in pg/mL for BeWo cells, while for villous explants, the data were normalized according to the protein concentration of each villous as described above. Then, for villous explants, the data about cytokines were obtained by the ratio between concentration of cytokines in pg/mL and concentration of total protein from Bradford assay in μg/mL, resulting in pg/μg of tissue. The limits of detection were determined from standard curves: IL-12p70, 7.8 pg/mL; IL-6, 4.7 pg/mL; TNF-α, 7.8 pg/mL; MIF, 7.8 pg/mL; IFN-γ, 4.7 pg/mL; IL-10, 7.8 pg/mL; and TGF-β1, 125 pg/mL.

### Parasite viability: trypan blue staining and transmission electron microscopy (TEM)

After verifying the toxicity, parasite intracellular proliferation, and cytokine production in BeWo cells and villous treated with enrofloxacin and toltrazuril, we wanted to investigate whether these drugs could trigger a direct effect in *T. gondii* tachyzoites or during division in host cells. For this purpose, we performed two different experiments.

Firstly, 1 × 10^6^ tachyzoites (2F1 clone) were added to microtubes in the absence of cells and treated with enrofloxacin (100 μg/mL) or toltrazuril (50 μg/mL) during 3 h in RPMI 1640 medium with 5% FBS at 37°C and 5% CO_2_. Next, the parasites were stained with Trypan blue. Viable tachyzoites (clear cytoplasm and negative trypan blue staining) and unviable parasites (dark cytoplasm and positive trypan blue staining) were counted under an optical microscope (Castanheira et al., 2015). Tachyzoites in microtubes treated with only medium were considered the control.

Secondly, BeWo cells (5 × 10^5^ cells/200 μL RPMI/24-well plates) were infected with *T. gondii* (2F1 clone, 5:1) and treated with enrofloxacin (100 μg/mL), toltrazuril (50 μg/mL), or medium (negative control) for 24 or 48 h. Next, the cells were fixed in Karnovsky solution containing 2% paraformaldehyde and glutaraldehyde in a 0.1 M sodium cacodylate buffer (pH 7.4). After, the cells were incubated for 1 h in 1% osmium tetroxide in cacodylate solution and treated with potassium ferrocyanide for an additional 30 min. Samples were dehydrated in increasing concentrations of ethanol, and embedded in propylene oxide and after in Epon resin. Ultrathin sections were stained with lead citrate and uranyl, and then analyzed in a FEI Tecnai G2 transmission electron microscope (TEM) (Hillsboro, Oregon, USA).

### Statistical analysis

Data were expressed as mean ± standard error of mean (SEM) of the experimental groups using GraphPad Prisma Software 5.0 (GraphPad Software, Inc., San Diego, CA, USA). Differences between groups were assessed by One-Way ANOVA with the Bonferroni multiple comparison *post-hoc* test, or Kruskall–Wallis with Dunn's multiple comparison *post-hoc* test, when appropriate. Statistical differences were considered significant when *P* < 0.05.

## Results

### Enrofloxacin and toltrazuril change the cellular viability in BeWo cells only at higher concentrations

The MTT assay was performed in order to evaluate the possible cytotoxicity of enrofloxacin, toltrazuril, sulfadiazine, pyrimethamine, combination of sulfadiazine plus pyrimethamine or only DMSO in BeWo cells.

**Figure 1:**
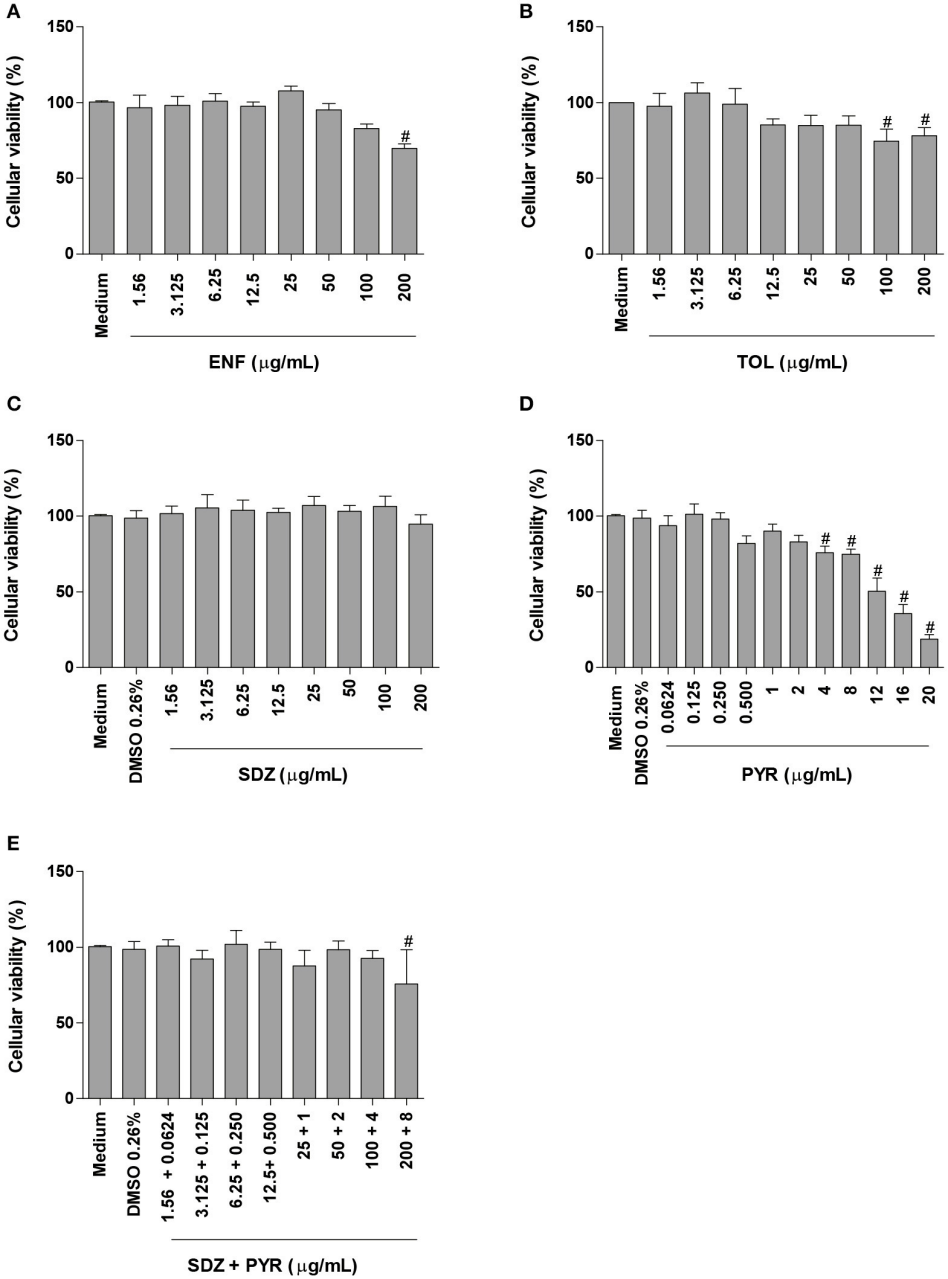
Cellular viability in BeWo cells treated with different drugs. BeWo cells were cultured in 96-well plates (3 × 10^4^ cells/well/200 μL) for 24 h and treated or not with DMSO (0.26%), enrofloxacin (ENF) **(A)**, toltrazuril (TOL) **(B)**, sulfadiazine (SDZ) **(C)**, pyrimethamine (PYR) **(D)**, or combination of sulfadiazine plus pyrimethamine (SDZ+PYR) **(E)** in different concentrations (μg/mL). After 24 h of treatment, the cells were submitted to MTT assay and data were presented as percentage (%) of viable cell (cellular viability) in relation to untreated cells (100% of cellular viability). Data were shown as mean ± SEM from three independents experiments with nine replicates. Significant differences in relation to untreated cells (medium) (^#^*P* < 0.05). Differences between groups were analyzed by One-Way ANOVA with the Bonferroni multiple comparison *post-hoc* test.

Enrofloxacin treatment reduced significantly the cellular viability only at high concentration (200 μg/mL) when compared to untreated cells (*P* < 0.05; Figure 1A). Toltrazuril significantly decreased the cellular viability at doses of 100 and 200 μg/mL in relation to untreated cells (*P* < 0.05; Figure 1B).

When BeWo cells were treated with sulfadiazine, there was no significant reduction in the cellular viability in comparison to untreated cells (Figure 1C). Treatment with pyrimethamine induced a significant reduction in the cellular viability at 4, 8, 12, 16, and 20 μg/mL, when compared to untreated cells (*P* < 0.05; Figure 1D). Combination of sulfadiazine plus pyrimethamine did not significantly reduce the viability of the cells when compared to untreated cells, except for 200 + 8 μg/mL (*P* < 0.05; Figure 1E). Also, treatment with medium plus DMSO (0.26%) only was not able to reduce the cellular viability in BeWo cells (Figures 1C–E).

Based on these data, the drug concentrations that did not change significantly the cellular viability were selected to carry out further experiments.

### Enrofloxacin and toltrazuril reduce the *T. gondii* intracellular proliferation in BeWo cells infected by highly virulent RH strain (2F1 clone)

To assess the effect of the selected drugs on *T. gondii* intracellular proliferation, infected BeWo cells were treated with them for 24 h and the β-galactosidase assay was performed.

Initially, we tested all low drug concentrations, and it was observed that these low doses were not able to control the *T. gondii* intracellular proliferation (data not shown). Next, we performed experiments using high concentrations that did not change the cell viability, except for pyrimethamine, since the doses that were not cytotoxic for this drug were unable to control the parasite. As result, BeWo cells treated with enrofloxacin (50 or 100 μg/mL) showed a significant reduction of the intracellular proliferation of the parasite when compared to untreated and infected cells (*P* < 0.05; Supplementary Figure 1A). In addition, 100 μg/mL enrofloxacin was more effective for control of the growth of the parasite in comparison with 50 μg/mL (*P* < 0.05; Supplementary Figure 1A). The treatments with toltrazuril reduced the *T. gondii* intracellular proliferation in all concentrations used in relation to untreated and infected cells (*P* < 0.05; Supplementary Figure 1B). The treatments with sulfadiazine or pyrimethamine decreased the parasite proliferation at all concentrations used in comparison to untreated and infected cells (*P* < 0.05; Supplementary Figures 1C,D). In addition, the combination of sulfadiazine and pyrimethamine also reduced the *T. gondii* intracellular proliferation at all doses used in comparison to untreated and infected cells (*P* < 0.05; Supplementary Figure 1E).

**Figure 2:**
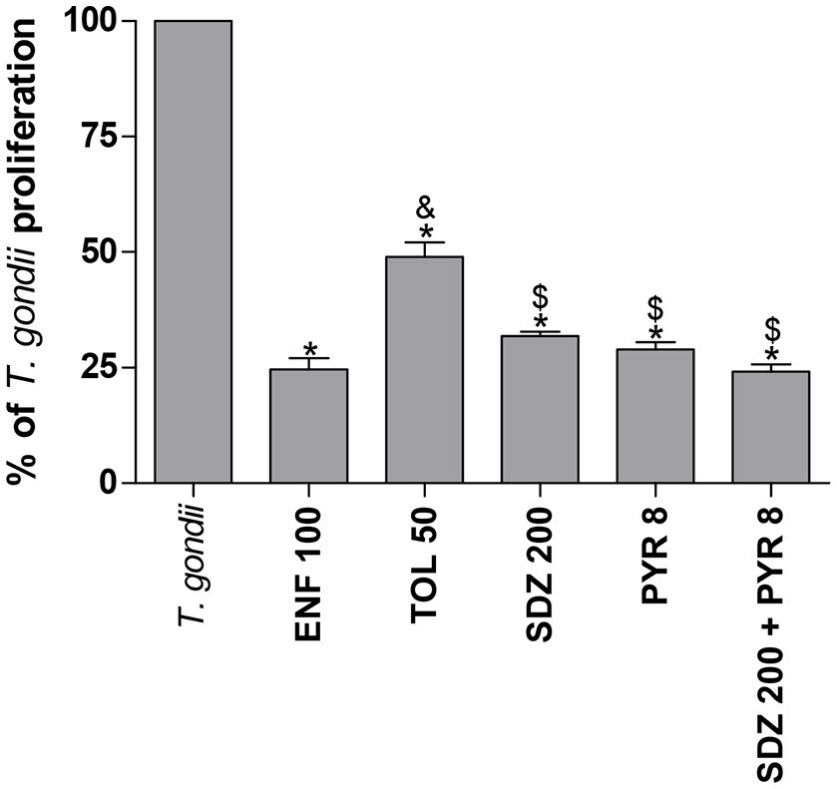
Percentage (%) of *T. gondii* proliferation (2F1 clone) in BeWo cells treated with different drugs. BeWo cells were cultured in 96-well plates (3 × 10^4^ cells/well/200 μL) for 24 h, infected with *T. gondii* (RH-2F1 clone) for 3 h, washed with medium to remove extracellular parasites and treated or not at the same time with different drugs (μg/mL): enrofloxacin (ENF), toltrazuril (TOL), sulfadiazine (SDZ), pyrimethamine (PYR), or combination of sulfadiazine and pyrimethamine (SDZ+PYR) for an additional 24 h. Next, the cells were submitted to *T. gondii* intracellular proliferation assay (β-galactosidase assay). Data were presented as mean ± SEM of the percentage (%) of *T. gondii* proliferation from three independent experiments in nine replicates. Significant differences in relation to infected and untreated cells (*T. gondii*) (^*^P < 0.05), ENF (^&^*P* < 0.05), and TOL (^$^*P* < 0.05). Differences between groups were analyzed by One-Way ANOVA with the Bonferroni multiple comparison *post-hoc* test.

To compare the effect of the different drugs, experiments using all drugs simultaneously were designed. Again, all of the drugs selected for this condition were able to significantly reduce the parasite proliferation in relation to untreated and infected cells (*P* < 0.05; Figure 2). However, enrofloxacin was more efficient to control the proliferation of *T. gondii* (around 75%) compared to toltrazuril (around 50%) (*P* < 0.05; Figure 2), although no significant difference was observed between enrofloxacin (75%), sulfadiazine (around 70%), pyrimethamine (around 70%), or combination of sulfadiazine plus pyrimethamine treatments (around 75%) (Figure 2). Finally, sulfadiazine, pyrimethamine or combination of sulfadiazine plus pyrimethamine treatments were more effective for the control of parasite proliferation than toltrazuril (*P* < 0.05; Figure 2). Thus, enrofloxacin and toltrazuril were both able to significantly reduce *T. gondii* intracellular proliferation.

### Enrofloxacin and toltrazuril reduce the *T. gondii* infection in BeWo cells infected by moderately virulent ME49 strain

Also, whether enrofloxacin and toltrazuril would be able to control *T. gondii* infection in the presence of a moderately virulent strain, ME49, was evaluated. For this purpose, the number of infected cells and the total number of intracellular tachyzoites were determined, with these data being expressed as infection index (percentage of infected cells) and *T. gondii* intracellular proliferation (total number of tachyzoites per 200 cells).

**Figure 3:**
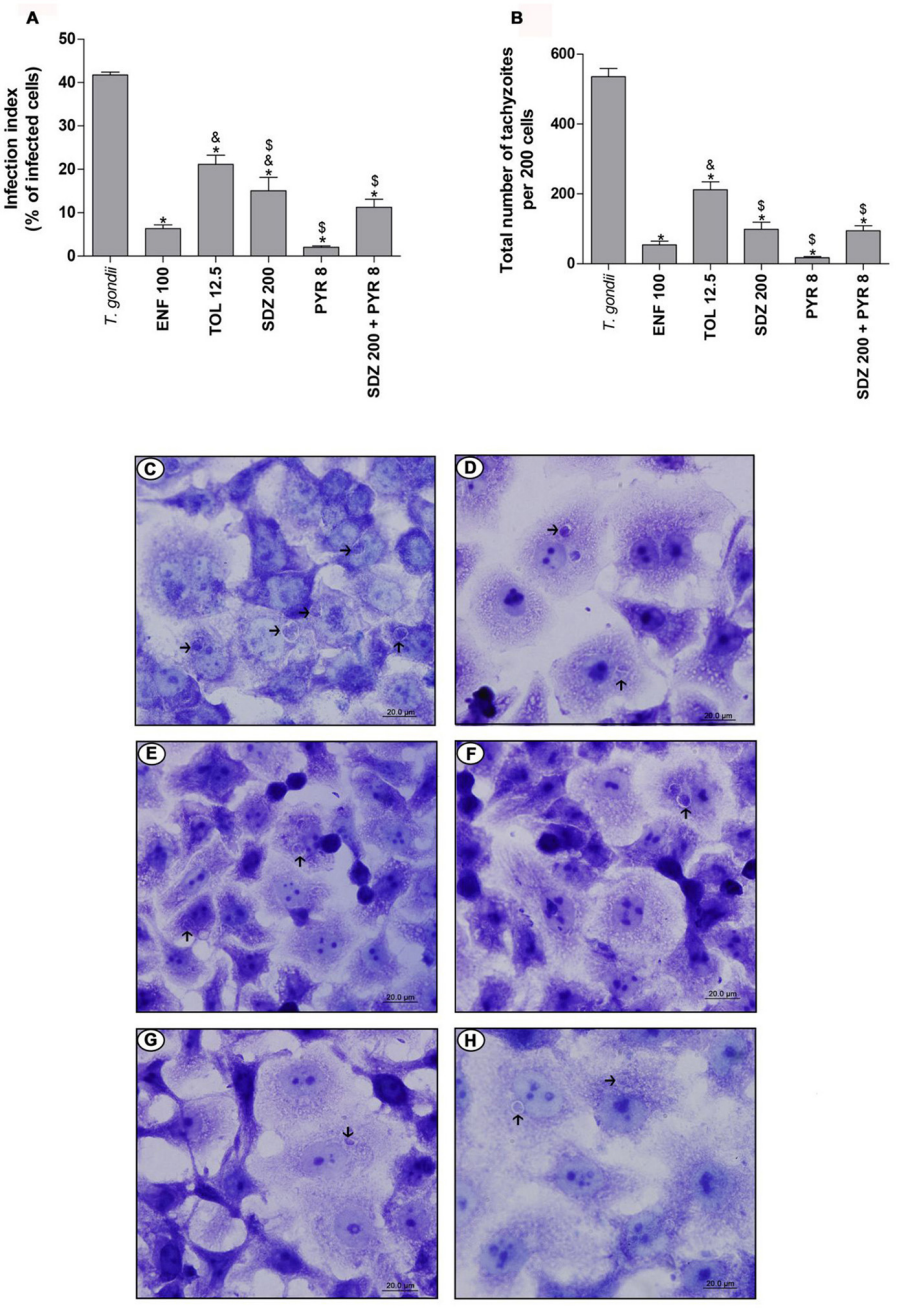
*T. gondii* infection (ME49 strain) in BeWo cells treated with different drugs. BeWo cells were cultured on round glass slides of 13 mm in 24-well plates (1 × 10^5^ cells/200 μL) for 24 h, infected with *T. gondii* (ME49 strain) for 3 h, washed with medium to remove extracellular parasites and treated or not with the different drugs (μg/mL): enrofloxacin (ENF), toltrazuril (TOL), sulfadiazine (SDZ), pyrimethamine (PYR), or combination of sulfadiazine plus pyrimethamine (SDZ+PYR) for an additional 24 h. Then, the cells were stained with 1% toluidine blue and counted using a light microscope. Data obtained were analyzed as infection index (% of infected cells) **(A)** and total number of tachyzoites (total number of tachyzoites per 200 cells) **(B)**. Data were shown as mean ± SEM from three independents experiments in six replicates. Significant difference in relation to infected and untreated cells (*T. gondii*) (^*^P < 0.05), ENF (^&^*P* < 0.05), and TOL (^$^*P* < 0.05). Differences between groups were analyzed by One-Way ANOVA with the Bonferroni multiple comparison *post-hoc* test. Representative photomicrographs of BeWo cells infected by *T. gondii* and untreated **(C)**, or treated with ENF **(D)**, TOL **(E)**, SDZ **(F)**, PYR **(G)**, or combination of SDZ+PYR **(H)**. Staining by blue toluidine. Arrows indicate parasites into parasitophorous vacuole. Bar scale: 20.0 μm.

The treatment with enrofloxacin significantly reduced the percentage of infected cells when compared to untreated and infected cells, and toltrazuril or sulfadiazine-treated cells (*P* < 0.05; Figure 3A). However, there were no significant differences between enrofloxacin and treatments with pyrimethamine or combination of sulfadiazine plus pyrimethamine (Figure 3A). The treatment with toltrazuril decreased the percentage of infected cells when compared to untreated and infected cells (Figure 3A), but toltrazuril was less effective for control of the infection index when compared to enrofloxacin, sulfadiazine, pyrimethamine, or combination of sulfadiazine and pyrimethamine treatments (*P* < 0.05; Figure 3A). For this experiment, we used 12.5 μg/mL toltrazuril since it was a sufficient dose to control *T. gondii* tachyzoites from ME49. In addition, sulfadiazine, pyrimethamine, and combination of sulfadiazine plus pyrimethamine reduced the percentage of infected cells in comparison to untreated and infected cells (*P* < 0.05; Figure 3A).

In relation to the total number of tachyzoites per 200 cells, treatment with enrofloxacin significantly reduced the *T. gondii* proliferation in BeWo cells when compared to untreated and infected cells and toltrazuril-treated cells (*P* < 0.05; Figure 3B); however, there was no significant difference in comparison to cells treated with sulfadiazine, pyrimethamine or combination of sulfadiazine plus pyrimethamine (Figure 3B). Toltrazuril significantly decreased the number of intracellular parasites when compared to untreated and infected cells, but it was less efficient for the control of parasite proliferation in relation to treatments with enrofloxacin, sulfadiazine, pyrimethamine, or combination of sulfadiazine plus pyrimethamine (*P* < 0.05; Figure 3B). Treatments with sulfadiazine, pyrimethamine, or combination of sulfadiazine plus pyrimethamine also reduced the *T. gondii* proliferation when compared to untreated and infected cells (*P* < 0.05; Figure 3B).

Representative photomicrographs are shown in Figures 3C–H, where it was possible to observe a higher number of parasitophorous vacuoles (arrows) in untreated and infected cells (Figure 3C) in comparison to enrofloxacin (Figure 3D), toltrazuril (Figure 3E), sulfadiazine (Figure 3F), pyrimethamine (Figure 3G), or combination of sulfadiazine and pyrimethamine (Figure 3H) treatments. Thus, enrofloxacin and toltrazuril were both able to significantly reduce the infection index and the total number of tachyzoites in BeWo cells infected with the ME49 strain; however, enrofloxacin was more efficient for the control of infection than toltrazuril and sulfadiazine.

### BeWo cells infected by *T. gondii* (RH-2F1 clone or ME49) and treated with enrofloxacin or toltrazuril produce IL-6 and MIF

After evaluating the control of *T. gondii* intracellular proliferation in BeWo cells treated with enrofloxacin, toltrazuril, sulfadiazine, pyrimethamine, or combination of sulfadiazine plus pyrimethamine, it was determined in cell supernatants whether the treatments affected the profile of cytokine release. For this purpose, we measured the cytokine levels in cells infected by *T. gondii* or not. It is important to emphasize that uninfected/treated BeWo cells and the respective control (medium) or 2F1-infected/treated cells and the respective control (medium) were cultured in 96-well plates, while ME49-infected/treated cells and the respective control (medium) were cultured in 24-well plates. Then, the comparisons were performed within each experimental group in relation to its respective control (negative control performed in the same plate—medium), with no comparisons made between the RH and ME49 strains or between different culture plate type.

**Figure 4:**
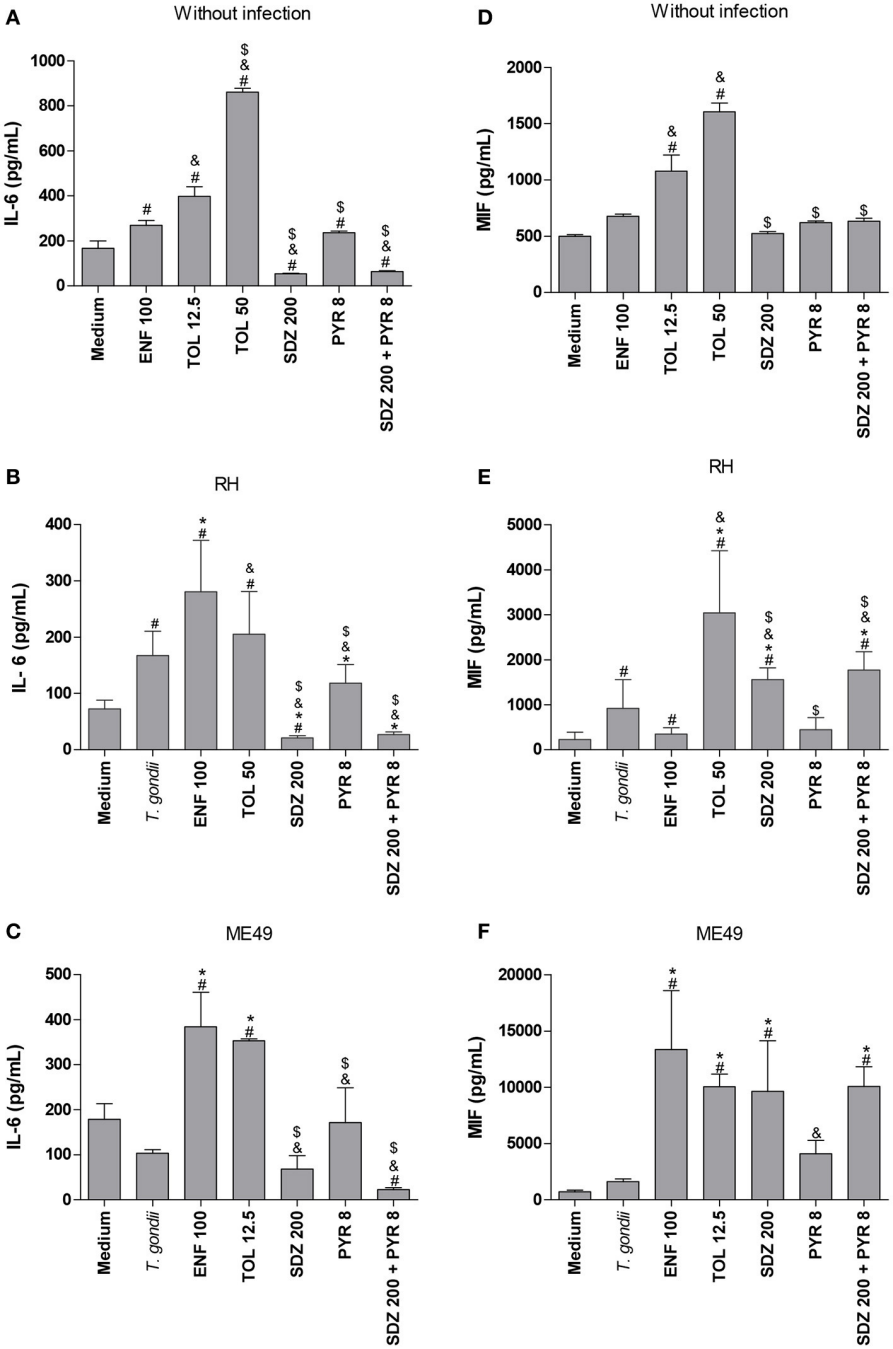
Cytokines production in BeWo cells treated with different drugs and infected by *T. gondii* (RH-2F1 clone or ME49 strain). BeWo cells were maintained in culture plates for 24 h, infected or not with *T. gondii* (RH-2F1 clone or ME49 strain) for 3 h, washed with medium to remove extracellular parasites and treated or not with the different drugs (μg/mL): enrofloxacin (ENF), toltrazuril (TOL), sulfadiazine (SDZ), pyrimethamine (PYR), or combination of sulfadiazine and pyrimethamine (SDZ+PYR) for additional 24 h. The supernatants were collected for measurement of IL-6 **(A–C)** and MIF **(D–F)** by ELISA. Uninfected/treated cells and the respective control (medium) **(A,D)** or 2F1-infected/treated cells and the respective control (medium) **(B,E)** were cultured in 96-well plates (3 × 10^4^ cells/well/200 μL), while ME49-infected/treated cells and the respective control (medium) **(C,F)** were cultured in 24-well plates (1 × 10^5^ cells/well/200 μL). Data were expressed as pg/mL according to the standard curve. Data were shown as mean ± SEM from three independent experiments in nine (2F1 clone) or six replicates (ME49). Significant difference in relation to untreated and uninfected cells (medium) (^#^*P* < 0.05), untreated and infected cells (*T. gondii*) (^*^P < 0.05), ENF (^&^*P* < 0.05), and TOL (^$^*P* < 0.05). Differences between groups were analyzed by One-Way ANOVA with the Bonferroni multiple comparison *post-hoc* test.

In the absence of infection, it was observed that enrofloxacin and toltrazuril increased IL-6 production compared to untreated cells (*P* < 0.05; Figure 4A). Additionally, toltrazuril augmented the IL-6 release in a dose-dependent manner, since 50 μg/mL induced higher IL-6 levels than 12.5 μg/mL (*P* < 0.05; Figure 4A). In addition, all toltrazuril concentrations induced higher IL-6 secretion than enrofloxacin-treated cells (*P* < 0.05; Figure 4A). Finally, sulfadiazine, pyrimethamine, and combination of sulfadiazine plus pyrimethamine reduced IL-6 production in comparison to untreated and toltrazuril-treated cells, while only sulfadiazine and combination of sulfadiazine plus pyrimethamine diminished IL-6 release in relation to enrofloxacin-treated cells (*P* < 0.05; Figure 4A).

In the presence of infection by RH strain, untreated cells presented high IL-6 release compared to untreated and uninfected cells (*P* < 0.05; Figure 4B). However, when ME49 tachyzoites were added to cells, no significant change in IL-6 production was observed in relation to untreated and uninfected cells (Figure 4C). Interestingly, cells treated with enrofloxacin, regardless of the *T. gondii* strain, augmented their IL-6 production in comparison to untreated and uninfected or only infected cells (*P* < 0.05; Figures 4B,C). Cells infected by RH and treated with toltrazuril increased the IL-6 secretion in relation to untreated and uninfected cells (*P* < 0.05; Figure 4B), but it was not significant in comparison to untreated and infected cells (Figure 4B). Toltrazuril induced high IL-6 secretion in cells infected by ME49 strain in relation to untreated and uninfected or only infected cells (*P* < 0.05; Figure 4C). Finally, cells infected with RH and treated with sulfadiazine, pyrimethamine, or combination of sulfadiazine plus pyrimethamine significantly diminished IL-6 production compared to untreated and infected cells or enrofloxacin- or toltrazuril-treated cells (*P* < 0.05; Figure 4B). Also, cells infected by ME49 and treated with sulfadiazine, pyrimethamine, or combination of sulfadiazine plus pyrimethamine reduced IL-6 release in relation to enrofloxacin- or toltrazuril-treated cells (*P* < 0.05; Figure 4C). Finally, BeWo cells infected by *T. gondii* (RH) and treated with sulfadiazine, or infected by ME49 strain and treated with combination of sulfadiazine plus pyrimethamine reduced IL-6 production in relation to untreated and uninfected cells (*P* < 0.05; Figures 4B,C).

Concerning MIF production, uninfected BeWo cells and those treated with enrofloxacin did not show a significant change in MIF compared to untreated cells (Figure 4D). However, all doses of toltrazuril induced high levels of MIF in comparison to untreated or enrofloxacin-treated cells (*P* < 0.05; Figure 4D). Sulfadiazine, pyrimethamine and combination of sulfadiazine plus pyrimethamine showed no significant difference in MIF release in relation to untreated cells, but it was lower than toltrazuril-treated cells (*P* < 0.05; Figure 4D).

When BeWo cells were only infected by tachyzoites from the RH strain, MIF production was increased in relation to untreated and uninfected cells (*P* < 0.05; Figure 4E). Also, enrofloxacin treatment in infected BeWo cells (RH) augmented MIF production compared to untreated and uninfected cells (*P* < 0.05; Figure 4E), although there was no difference in relation to untreated and infected cells (Figure 4E). Toltrazuril, sulfadiazine or combination of sulfadiazine plus pyrimethamine treatments induced high MIF levels in comparison to untreated and uninfected or only infected or enrofloxacin-treated cells (*P* < 0.05; Figure 4E). Pyrimethamine did not significantly alter the MIF levels when compared to untreated cells, regardless of the infection (Figure 4E). Sulfadiazine, pyrimethamine, or combination of sulfadiazine plus pyrimethamine induced lower MIF levels in infected cells in comparison to toltrazuril-treated cells (*P* < 0.05; Figure 4E). Concerning ME49 infection and MIF production, infected and untreated BeWo cells did not show a significant difference in relation to untreated and uninfected cells (Figure 4F). However, enrofloxacin, toltrazuril, sulfadiazine and combination of sulfadiazine plus pyrimethamine treatments induced an increase in MIF release in comparison to untreated and uninfected or only infected cells (*P* < 0.05; Figure 4F). Finally, pyrimethamine did not induce a significant change in MIF levels in relation to untreated and uninfected or infected cells (Figure 4F).

TGF-β1, IL-12p70, TNF-α, IL-10, and IFN-γ cytokines were not detected in BeWo cells under any experimental conditions (data not shown).

### Enrofloxacin and toltrazuril are not toxic to villous explants, even at high concentrations

After evaluating the influence of enrofloxacin and toltrazuril in BeWo cells infected by RH or ME49 strains, the effects of the selected drug panel against *T. gondii* infection was analyzed in human villous explants. To design these new experiments, the parasite 2F1 clone was chosen for three reasons: (i) both drugs were able to control the parasitism in BeWo cells, regardless of the parasite strain (RH or ME49); (ii) RH is a more virulent strain than ME49; and (iii) the RH strain was discovered/isolated from a newborn with congenital toxoplasmosis, with many cases of congenital toxoplasmosis having been reported with type I strain (Fuentes et al., 2001).

**Figure 5:**
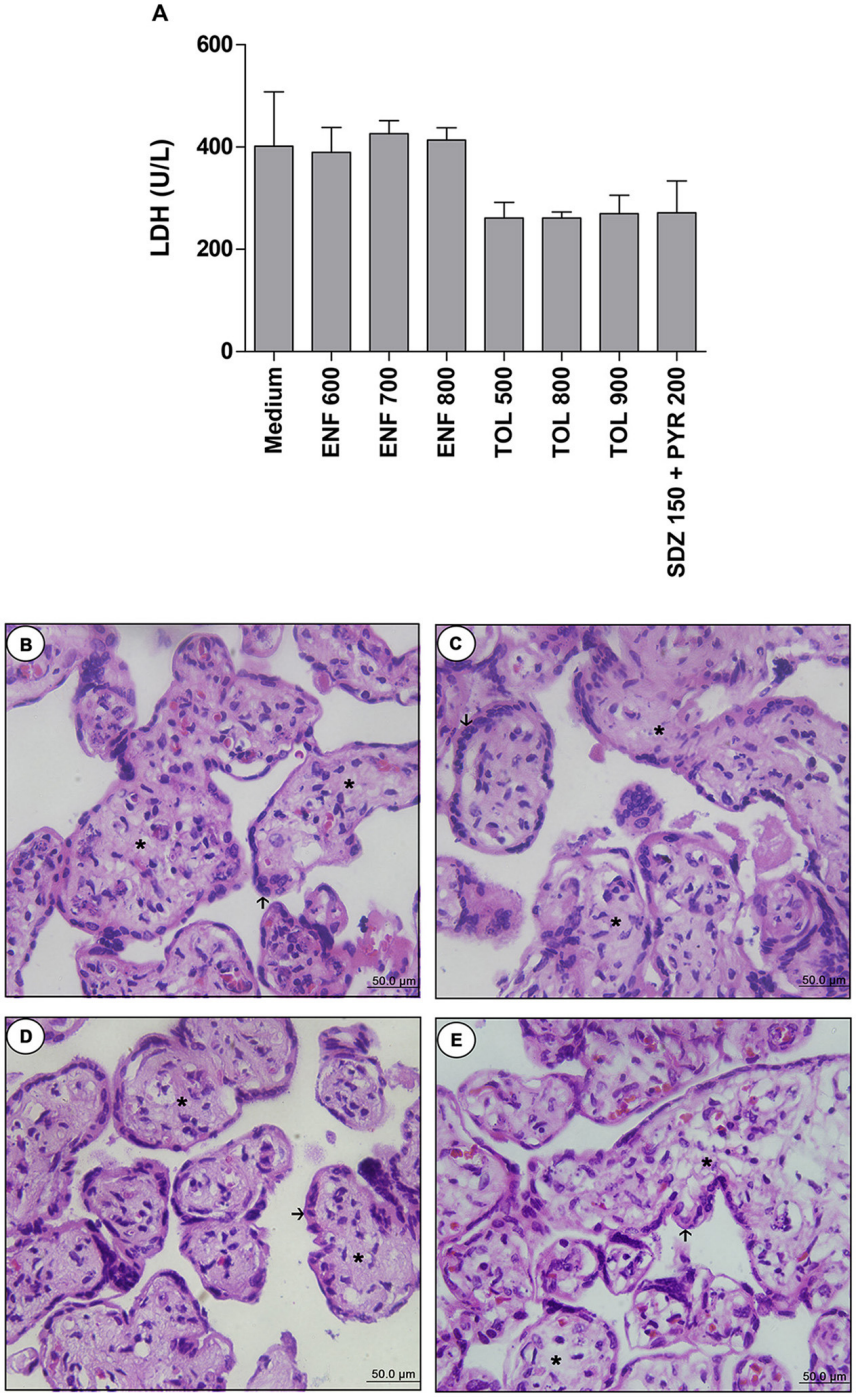
Analysis of toxicity in human villous explants treated with different drugs.Villous explants were cultured during 24 h in 96-well plates and treated or not with different drugs (μg/mL): enrofloxacin (ENF), toltrazuril (TOL) or combination of sulfadiazine plus pyrimethamine (SDZ+PYR). After 24 h of treatment, supernatants were collected and lactate dehydrogenase (LDH) activity was measured using the LDH Liquiform Kit **(A)**. Negative control was represented by villous treated with only RPMI medium. Data were expressed as mean ± SEM from three independent experiments in five replicates. Significant differences in relation to negative control (medium) (^#^*P* < 0.05). Differences between groups were analyzed by One-Way ANOVA with the Kruskall Wallis Dunn's multiple comparison *post-hoc* test. Representative photomicrographs of untreated villous explants **(B)**, or treated with ENF (700 μg/mL) **(C)**, TOL (900 μg/mL) **(D)**, or combination of SDZ+PYR (150 + 200 μg/mL) **(E)**. Histological sections stained by hematoxylin and eosin. Arrows indicate the syncytiotrophoblast cells and asterisks(^*^) the mesenchyme. Bar scale: 50.0 μm.

Firstly, we performed the viability assay in the villous. According to LDH measurement, treatments with all concentrations of enrofloxacin, toltrazuril, or combination of sulfadiazine plus pyrimethamine did not alter the tissue viability when compared to the negative control (medium, 100% viability) (Figure 5A). When the tissue morphology was analyzed, enrofloxacin, toltrazuril, or combination of sulfadiazine and pyrimethamine maintained the tissue structure, presenting normal morphology of the syncytiotrophoblast cells (arrows) and mesenchyme (asterisk) when compared to untreated villous (Figures 5B–E). Then, based on the data related to toxicity for villous explants, the concentrations of 700 and 900 μg/mL were chosen for enrofloxacin and toltrazuril, respectively, for further experiments.

### Enrofloxacin and toltrazuril are more efficient to control the *T. gondii* proliferation than sulfadiazine and pyrimethamine in human villous explants

**Figure 6:**
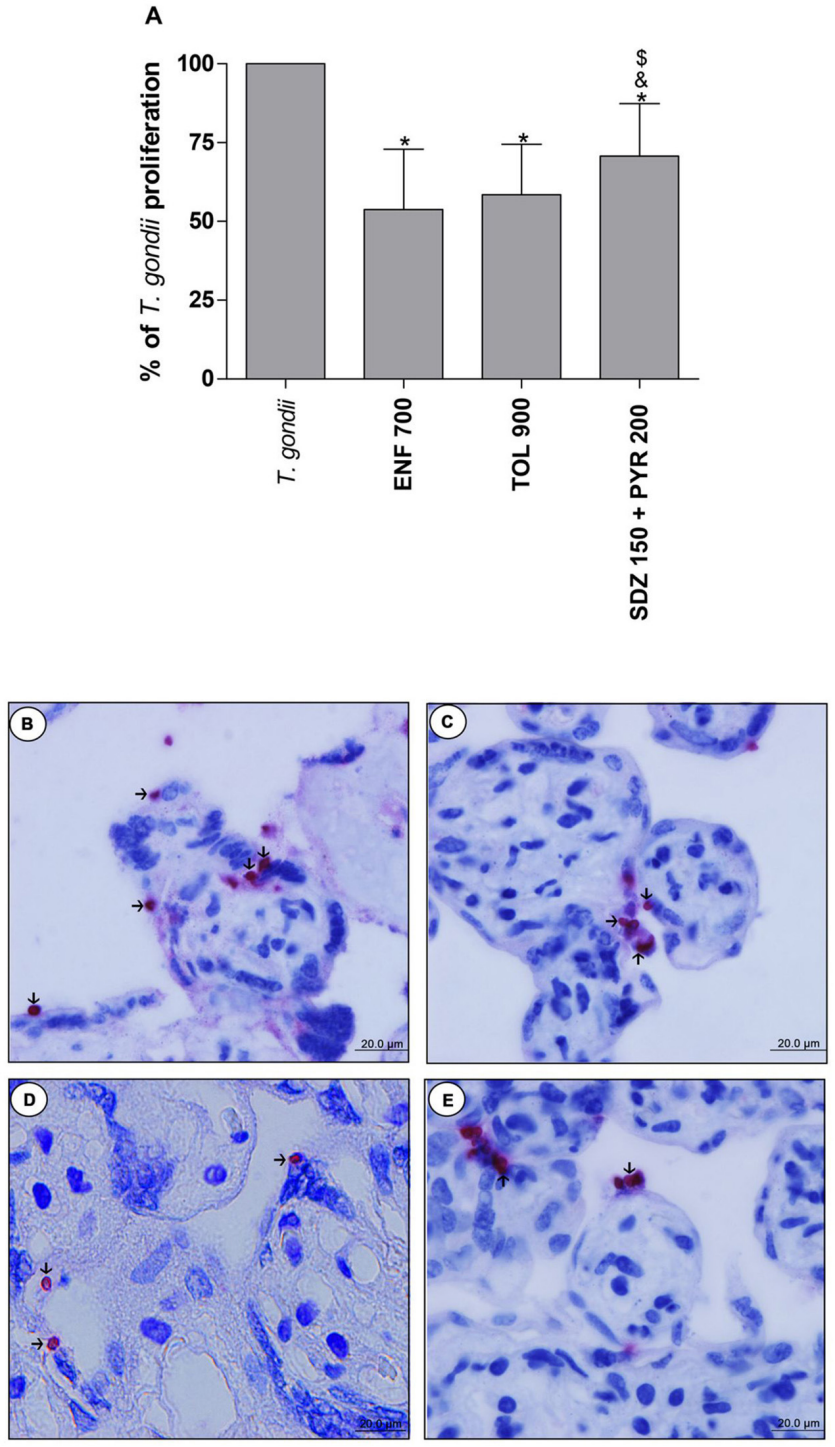
Percentage (%) of *T. gondii* proliferation in human villous explants treated with different drugs. Villi were collected and cultured for 24 h, infected with *T. gondii* (RH-2F1 clone), and after 24 h, treated or not with the different drugs (μg/mL): enrofloxacin (ENF), toltrazuril (TOL), or combination of sulfadiazine and pyrimethamine (SDZ+PYR) for additional 24 h. Next, the villous explants were macerated for β-galactosidase assay.Data were analyzed as (%) of *T. gondii* proliferation **(A)**. Data were expressed as mean ± SEM of three independent experiments in nine replicates. Significant difference in relation to untreated and infected cells (*T. gondii*) (^*^P < 0.05), ENF (^&^*P* < 0.05), and TOL (^$^*P* < 0.05). Differences between groups were analyzed by One-Way ANOVA with the Bonferroni multiple comparison *post-hoc* test. Representative photomicrographs of untreated and infected villous explants **(B)**, or treated with ENF **(C)**, TOL **(D)**, or combination of SDZ+PYR **(E)**. Immunohistochemical sections counterstained by Harris's hematoxylin. Arrows indicate immunolocalization of parasites by fast red naphtol. Bar scale: 20.0 μm.

The analysis of *T. gondii* intracellular proliferation in villous explants was performed by β-galactosidase assay. Enrofloxacin, toltrazuril and combination of sulfadiazine plus pyrimethamine decreased the *T. gondii* proliferation when compared to untreated and infected villous (*P* < 0.05; Figure 6A). Furthermore, enrofloxacin and toltrazuril were more efficient to control parasite proliferation in comparison to combination of sulfadiazine plus pyrimethamine treatment (*P* < 0.05; Figure 6A).

Representative photomicrographs are evidenced in Figures 6B–E, where it was possible to observe higher number of tachyzoites (arrows) in untreated and infected villi (Figure 6B), in comparison to enrofloxacin (Figure 6C), toltrazuril (Figure 6D), or combination of sulfadiazine plus pyrimethamine (Figure 6E) treatments. Once again, it was possible to observe no change in the tissue structure, presenting normal morphology of the syncytiotrophoblast cells and mesenchyme when compared to untreated villous (Figures 6B–E).

### Enrofloxacin induces MIF release in human villous explants

**Figure 7:**
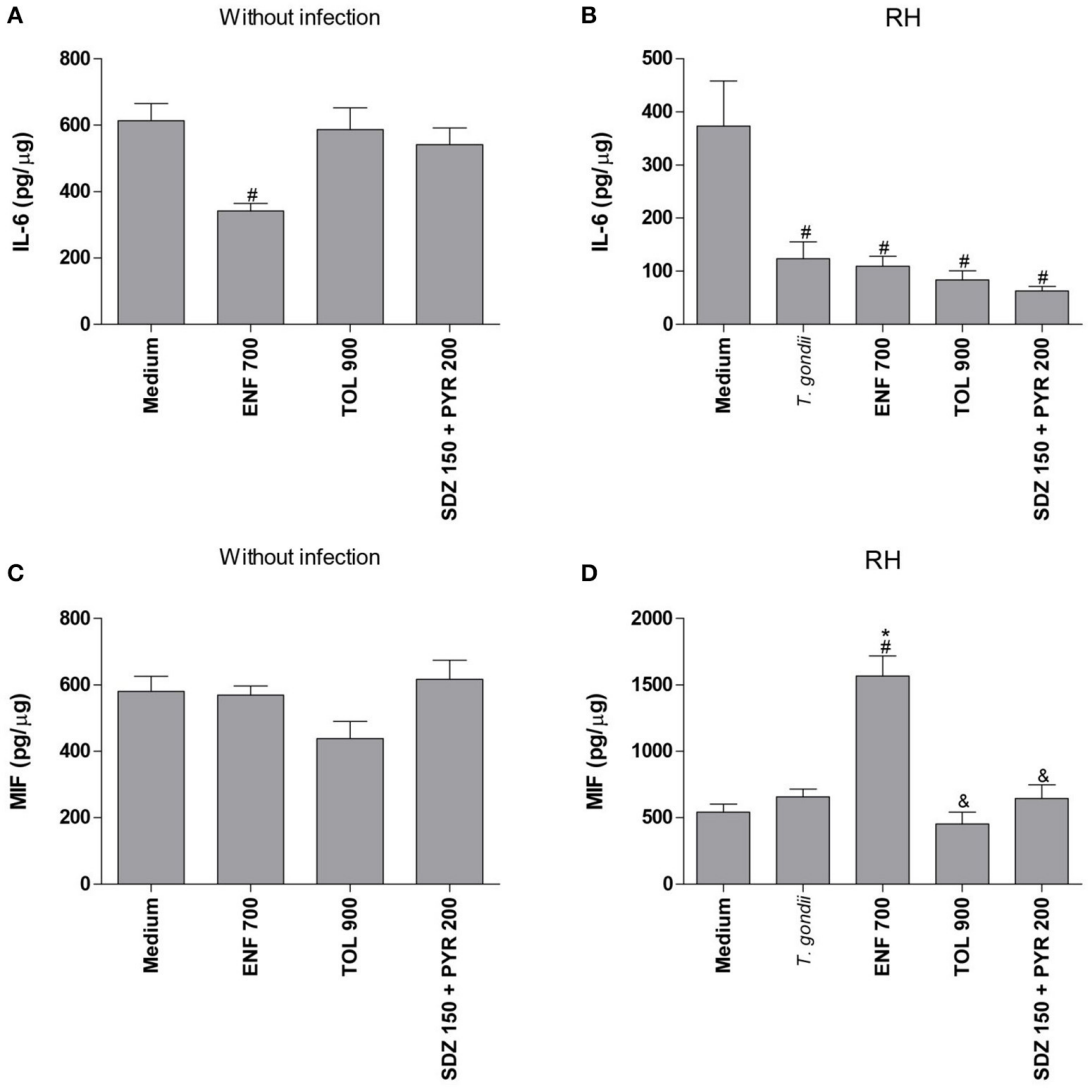
Cytokines production in human villous explants treated with different drugs and infected by *T. gondii*. Villous explants were collected and cultured during 24 h, infected or not with *T. gondii* (RH-2F1 clone), and after 24 h were treated or not with the different drugs (μg/mL): enrofloxacin (ENF), toltrazuril (TOL) or combination of sulfadiazine and pyrimethamine (SDZ+PYR) for additional 24 h.The supernatants were collected for detection of IL-6 **(A,B)** and MIF **(C,D)**. Data were expressed in pg/μg according to the protein concentration of each villous and the standard curve. Data were expressed as mean ± SEM of three independent experiments in nine replicates. Significant difference in relation to untreated and uninfected villous (medium) (^#^*P* < 0.05), untreated and infected villous (*T. gondii*) (^*^P < 0.05), and ENF (^&^*P* < 0.05). Differences between groups were analyzed by One-Way ANOVA with the Bonferroni multiple comparison *post-hoc* test.

After analyzing the *T. gondii* proliferation in villous explants infected and treated with different drugs, the profile of cytokines in the absence or presence of infection was determined (Figure 7).

In the absence of *T. gondii*, enrofloxacin downmodulated IL-6 production (*P* < 0.05), but no significant change in MIF release was detected when compared to untreated and uninfected villous (Figures 7A,C). On the other hand, toltrazuril or combination of sulfadiazine plus pyrimethamine treatments did not alter IL-6 or MIF production in relation to untreated and uninfected villi (Figures 7A,C).

It was observed that *T. gondii* infection promoted a significant reduction of IL-6 production in untreated villous explants in comparison to untreated and uninfected villi (*P* < 0.05; Figure 7B). The MIF production was not changed by the parasite in untreated villous (Figure 7D). When enrofloxacin, toltrazuril or combination of sulfadiazine plus pyrimethamine were added in the infected villous, there was a reduction of IL-6 production when compared to untreated and uninfected villi (*P* < 0.05); however, no statistically significant difference was observed in relation to untreated and infected villi (Figure 7B), which demonstrated that the effect of the downmodulation of IL-6 is due to the presence of the parasite. Interestingly, enrofloxacin treatment induced an upregulation of MIF production when compared to untreated/uninfected or untreated/infected villous (*P* < 0.05; Figure 7D). The remaining treatments did not promote any effect on MIF modulation (Figure 7D).

TGF-β1, IL-12p70, TNF-α, IL-10, and IFN-γ cytokines were not produced in villous explants under any experimental conditions (data not shown).

### Enrofloxacin and toltrazuril reduce the parasite viability and promote damages during tachyzoite cell division

Finally, we investigated the direct effect of enrofloxacin and toltrazuril in *T. gondii* tachyzoites viability, as well as the effects of these drugs on infected BeWo cells, since the ultra-structure of treated intracellular parasites was observed by TEM.

**Figure 8:**
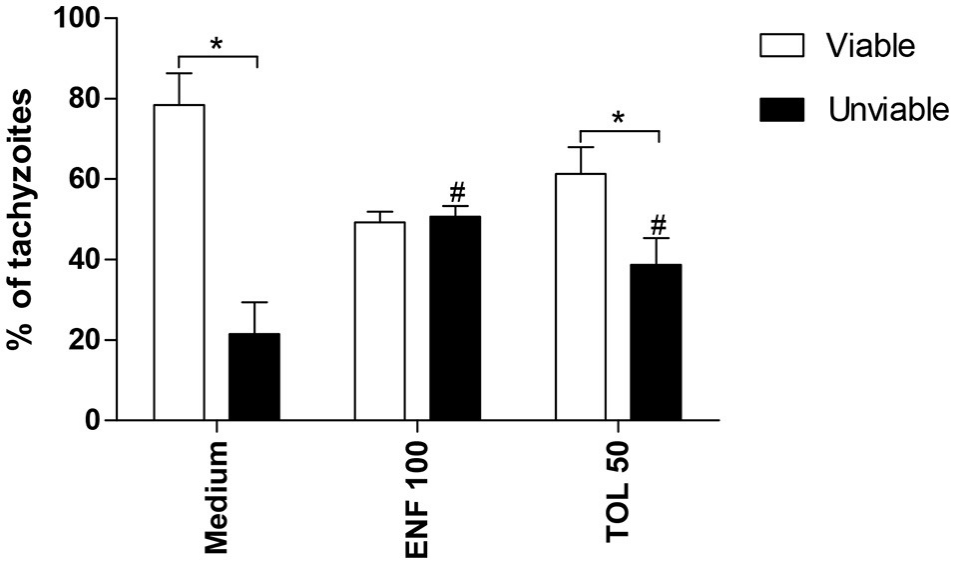
Viability of *T. gondii* tachyzoites. Tachyzoites were placed in microtubes (1 × 10^6^) and treated (μg/mL) or not with enrofloxacin (ENF), toltrazuril (TOL) or medium only for 3 h. After, the parasites were stained with trypan blue and counted in an optical microscope. Significant differences between viable and unviable parasites from the same group (^*^P < 0.05), and between unviable parasites from the treated and untreated-group (^#^*P* < 0.05).

The percentage of viable tachyzoites was higher in untreated (medium) or toltrazuril-treated parasites in relation to unviable parasites (*P* < 0.05). However, the percentage of unviable parasites increased significantly when enrofloxacin or toltrazuril were used to treat the tachyzoites in comparison to untreated parasites (medium) (*P* < 0.05; Figure 8).

**Figure 9:**
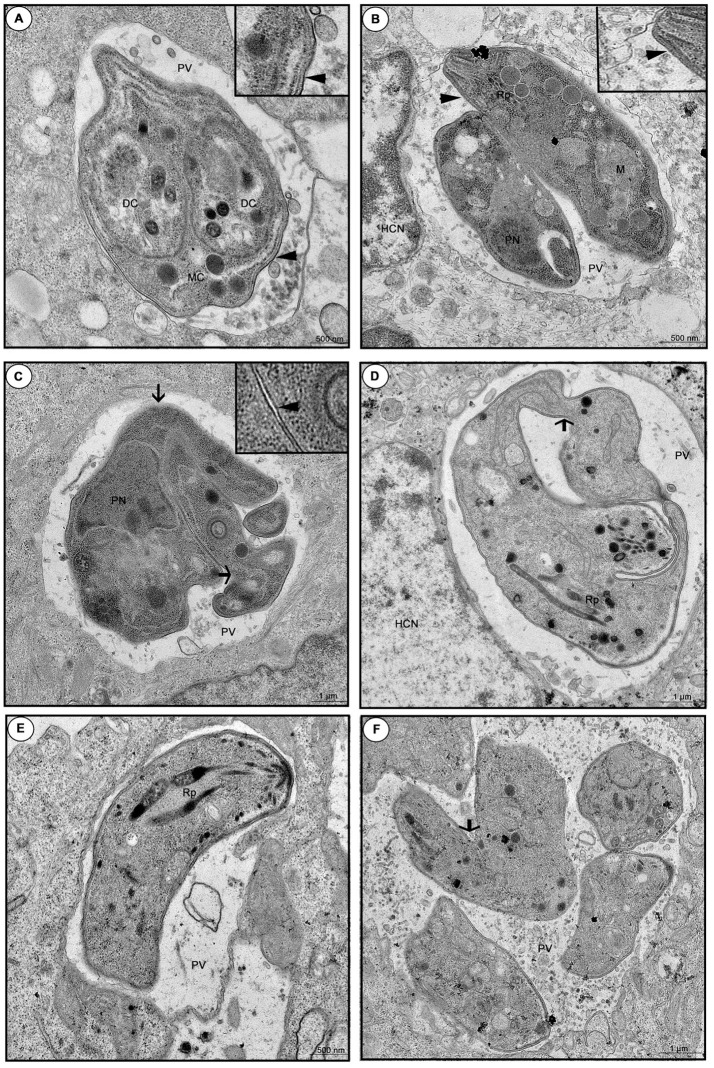
Ultra-structural analyze of tachyzoites in BeWo cells treated with enrofloxacin or toltrazuril. BeWo cells were cultured in 24-well plates (5 × 10^5^ cells/well/200 μL) for 24 h, infected with *T. gondii* (RH-2F1 clone) for 3 h, washed with medium to remove extracellular parasites and treated (μg/mL) or not with enrofloxacin or toltrazuril for an additional 24 or 48 h.Then, the cells were submitted to transmission electron microscopy. Representative electromicrography of untreated BeWo cells infected by *T. gondii* by 24 **(A)** or 48 h **(B)**, enrofloxacin-treated cells infected by *T. gondii* by 24 **(C)** or 48 h **(D)**, and toltrazuril-treated cells infected by *T. gondii* by 24 **(E)** or 48 h **(F)**. PV, Parasitophorous vacuole; DC, daughter cells; MC, mother cell; HCN, host cell nucleus; Rp, rhoptries; PN, nucleus; and M, mitochondria of the parasites. Arrows: basal ends (“tethered” parasites); arrowhead: duple membrane. Bar scale: 500 nm or 1.0 μm.

When we investigated the ultra-structure by TEM during 24 or 48 h infection, untreated cells showed a parasitophorous vacuole (PV) containing tachyzoites in a normal cell division, since daughter cells (DC) were transformed into mother cells (MC), via a classical endodiogeny process (Figure 9A). Furthermore, it is possible to observe the duple membrane (arrowheads), rhoptries (Rp), nucleus (PN), and mitochondria (M) of the parasites, confirming the morphology of a typical viable parasite (Figures 9A,B). On the other hand, enrofloxacin-treated infected cells demonstrated PV containing “tethered” tachyzoites, since the treatment triggered budding arrest; also, we frequently detected parasites united by their basal ends (arrows), regardless of the time of infection, 24 or 48 h, probably due to a difficulty in completing cytokinesis (Figures 9C,D). Additionally, it was possible to observe a single membrane in some points of the parasites, suggesting damage to the formation of the duple membrane (arrowhead) (Figure 9C). Finally, toltrazuril did not induce a significant change in the ultra-structure (Figure 9E), but some “tethered” parasites (arrow) were observed, although these were at a low frequency compared to enrofloxacin, regardless of the time of infection, 24 or 48 h (Figure 9F). We also verified the *T. gondii* intracellular proliferation in BeWo cells infected and treated for 48 h by beta-galactosidase assay, and it was detected that enrofloxacin and toltrazuril significantly reduced the *T. gondii* growth (data not shown), as observed for 24 h.

## Discussion

In the present study, we evaluated the effect of enrofloxacin and toltrazuril in the control of *T. gondii* infection in BeWo cells and human villous explants from third trimester placentas.

At first, the effect of enrofloxacin and toltrazuril in BeWo cells was analyzed to determine the best concentrations of the drugs to inhibit parasite growth, without being cytotoxic to host cells. Pyrimethamine, a classical treatment, significantly reduced the cellular viability in a dose-dependent manner. However, enrofloxacin and toltrazuril did not significantly reduce the cellular viability in BeWo cells, maintaining more than 80% of the host cells as viable, except for 200 μg/mL enrofloxacin and 100 or 200 μg/mL toltrazuril. Then, this is the first study to demonstrate the tolerance of human trophoblastic cells (BeWo line) to enrofloxacin or toltrazuril treatments, even at higher doses in comparison to pyrimethamine. Our present data agree with our previous study, which also observed that enrofloxacin diminished slightly the cellular viability in human fibroblast cells (HFF) (Barbosa et al., 2012). On the other hand, treatment with high doses of toltrazuril in HFF cells may promote cytotoxicity in these cells, since only low concentrations are able to maintain 80% viability (Qian et al., 2015). These data suggest that different cellular populations respond distinctly to different drug types. A toxic effect of pyrimethamine was observed, even at low doses, in human trophoblastic cells, emphasizing the importance of studies concerning new therapies to treat or minimize the severe effects triggered by the vertical transmission of *T. gondii*.

Additionally, when the toxicity was analyzed in human villous explants, the viability was maintained for all types of treatment tested, as demonstrated by the LDH assay and, particularly, by morphological analyses. A previous study showed that analysis of the morphology of cultured villous explants by light microscopy is one of the most important parameters of tissue viability *in vitro*, considering that syncytiotrophoblasts are very susceptible to environmental conditions and, if something alters their viability, the cells in this compartment are the first to be degenerated, as it is clear to observe syncytial sloughing and detachment due to fibrinoid necrosis (Miller et al., 2005). A previous study by our group also showed that human villous explants were resistant to azithromycin treatment, as verified by the LDH assay (Castro-Filice et al., 2014). Thus, this is the first study to show no toxicity for human villous explants from the third trimester to enrofloxacin or toltrazuril treatments.

Next, the viability in BeWo cells and villous explants under treatment schedules with different drugs was investigated in the present study with focus on susceptibility to *T. gondii* infection. The results demonstrated that enrofloxacin and toltrazuril reduced the *T. gondii* intracellular proliferation in BeWo cells infected by RH or ME49 strains. Interestingly, enrofloxacin demonstrated higher effectiveness for control of the parasite proliferation by both strains compared to toltrazuril in BeWo cells. During ME49 infection, enrofloxacin was also more effective to reduce the *T. gondii* infection than sulfadiazine in BeWo cells. Even at increased doses, enrofloxacin (100 μg/mL) and toltrazuril (12.5 or 50 μg/mL) were not toxic to the cells and controlled the infection, while pyrimethamine was significantly cytotoxic at low doses (4–20 μg/mL), reducing the *T. gondii* proliferation only at these cytotoxic doses. Then, enrofloxacin and toltrazuril are good alternatives for the treatment or prevention of congenital toxoplasmosis, since high doses of these drugs did not alter the cellular viability and, at the same time, are able to control the parasite growth efficiently if compared to classical treatments.

In agreement with these data, a recent study by our group demonstrated that enrofloxacin is able to control *T. gondii* infection (RH and ME49 strain) in experimental models represented by HFF cells and the brains of *C. callosus* rodents (Barbosa et al., 2012). In addition, previous studies reported a protector effect of other fluoroquinolones against *T. gondii* proliferation *in vitro*, such trovafloxacin (Khan et al., 1996), gatifloxacin alone or in combination with pyrimethamine or IFN-γ (Khan et al., 2001), and ciprofloxacin (Dubar et al., 2011; Martins-Duarte et al., 2015). Only a few studies have approached the role of toltrazuril against *T. gondii* infection; and the results so far are not conclusive. Toltrazuril diminished the number of *T. gondii* cysts in skeletal musculature and brain from sheep by around 50% (Kul et al., 2013). A previous study evaluated the action of both enrofloxacin and toltrazuril to control the vertical transmission of *N. caninum* in C57/BL6 mice; it was found that both drugs were able to reduce congenital neosporosis, although toltrazuril presented increased efficacy in comparison to enrofloxacin (Gottstein et al., 2005). Therefore, although enrofloxacin and toltrazuril show a protective role against *T. gondii* for several cell types and tissues (Barbosa et al., 2012; Kul et al., 2013), to the best of our knowledge, the present study is the first to demonstrate the control of *T. gondii* proliferation by highly and moderately virulent strains in human trophoblastic cells.

When the parasite proliferation in villous explants was assessed, it was possible to observe similar results. Interestingly, both enrofloxacin and toltrazuril were more efficient for the control of *T. gondii* replication in relation to villi treated with classical drugs (combination of sulfadiazine plus pyrimethamine). There is only a single study in the literature demonstrating the effectiveness of alternative drugs against *T. gondii* in human villous explants; it was observed that azithromycin is also able to control *T. gondii* infection in human placenta (Castro-Filice et al., 2014). Although, high doses of enrofloxacin (600–800 μg/mL) and toltrazuril (500–900 μg/mL) have been used compared to combination of sulfadiazine plus pyrimethamine (150 + 200 μg/mL) in villous explants, these alternative drugs did not change the viability tissue. We tested lower doses of enrofloxacin and toltrazuril, but they are not effective for the control of parasite growth in villous explants (data not shown), which promoted the need to increase the concentrations of these drugs. Furthermore, in the present study, we did not investigate increased doses of combination of sulfadiazine plus pyrimethamine since they killed all of the placental tissue in previous studies by our group (Castro-Filice et al., 2014). This means that classical treatment is very toxic to placental tissues in elevated doses and did not guarantee the successful control of *T. gondii*. Thus, once again, enrofloxacin and toltrazuril, even at higher doses, were able to control parasite proliferation, since they did not induce toxicity and reduced the *T. gondii* infection in villous explants more efficiently in comparison to classical treatment.

In general, enrofloxacin and toltrazuril can be considered a good alternative treatment to prevent, minimize or treated the congenital toxoplasmosis for some reasons: (1) even at higher doses, there were no cytotoxic results, while classical treatment with pirymethamine induced increased cytotoxicity in low doses, (2) during ME49 infection (enrofloxacin) and in villous explants (both enrofloxacin and toltrazuril), the drugs were more effective for the control of parasites than classical treatment, and (3) previous studies have already shown that antibiotics from the fluoroquinolone group are not associated with teratogenic effects to the fetus (Larsen et al., 2001) and there was no report of complications in gestation during treatment with toltrazuril. Additionally, enrofloxacin and toltrazuril are already widely used for the treatment of infections caused by bacteria and coccidiosis in other animals. Due to the difficulty in discovering and developing new drugs that could be effective against *T. gondii* and other parasitic protozoa, some strategies were performed, such as expanding the application of existing drugs, with clinical safety, for the treatment of other diseases. Drug repurposing represents some advances as reduced costs, progress in clinical tests phases, an enhanced drug development process, the possibility of recuperating and repurposing some drugs that had failed before, and mainly safety (Andrews et al., 2014). Then, since enrofloxacin and toltrazuril are commonly used in veterinary medicine, and they are safe drugs, they have become a great alternative for the treatment of congenital toxoplasmosis, as drugs repositioning.

After the evaluation of *T. gondii* intracellular proliferation in BeWo cells and villous explants, the cytokine profile was analyzed in BeWo cells infected with type I or II strains, and in villous infected with type I strain. Data obtained from ELISA demonstrated that enrofloxacin and toltrazuril increased IL-6 and MIF production in BeWo cells infected by *T. gondii*, regardless of strain, while in the absence of infection, enrofloxacin induced only high IL-6 levels and toltrazuril triggered high IL-6 and MIF levels. In relation to villous explants, IL-6 was reduced in infected villous, regardless of treatments; however, the MIF production was increased when villous explants were treated with enrofloxacin. Thus, enrofloxacin and toltrazuril are able to act as modulators of the cytokine release by trophoblastic cells and villous explants, especially of IL-6 and MIF. The upregulation of these cytokines by both drugs is a potential mechanism triggered by trophoblastic cells to control the *T. gondii* infection.

IL-6 is a multifunctional cytokine secreted by trophoblastic cells and is associated with extensive biological functions, including inflammation, immune regulation, cellular differentiation, proliferation, migration, and apoptosis, all functions that are essential for normal placental development and successful pregnancy (Naka et al., 2002; Prins et al., 2012; Goyal et al., 2013). Several studies reported IL-6 to be pro-inflammatory, due to its role in protecting against some parasites, such as *T. gondii* (Mirpuri and Yarovinsky, 2012; Castro et al., 2013), including in BeWo trophoblastic cells (Barbosa et al., 2015). Also, the IL-6 secretion in BeWo cells was shown to be important in controlling the *T. gondii* infection in human monocytes (Castro et al., 2013). Additional studies have demonstrated an important protective function of IL-6 against other pathogens, such as *Trypanosoma cruzi* (Gao and Pereira, 2002) and *Giardia duodenalis* (Kamda et al., 2012). Thus, as it was previously demonstrated that IL-6 downmodulates the parasite proliferation in BeWo cells (Barbosa et al., 2015), it is possible to speculate that enrofloxacin or toltrazuril were able to control the *T. gondii* infection in BeWo cells since these antibiotics triggered IL-6 production by these cells. This cytokine was induced by both drugs, and probably reduced the parasite proliferation in BeWo cells infected by the RH or ME49 strain. However, it is not possible to exclude the direct effect of enrofloxacin on the tachyzoites structure; therefore, we investigated this hypothesis when we treated only the parasites or the infected cells to verify changes in tachyzoite structure.

MIF is also a pro-inflammatory cytokine involved in the adaptive and innate immune response (Kim et al., 2014). Studies demonstrated that MIF is produced by several cell types, such as macrophages, dendritic cells and lymphocytes (Murakami et al., 2002). Furthermore, MIF is an important cytokine in the immunophysiology of reproduction, since it is secreted by human trophoblast cells and human villous explants from the first and third trimester of gestation (Ferro et al., 2008; de Oliveira Gomes et al., 2011; Franco et al., 2011; Barbosa et al., 2014). In addition, MIF has a critical role during infection by many parasites, including *Leishmania major* (Satoskar et al., 2001) and *T. cruzi* (Reyes et al., 2006), and there have been previous studies emphasizing its importance against *T. gondii* infection in maternal-fetal interface (Ferro et al., 2008; Flores et al., 2008; de Oliveira Gomes et al., 2011; Barbosa et al., 2014). Thus, as it was previously demonstrated that MIF downmodulates the parasite proliferation in BeWo cells (Barbosa et al., 2014), is possible to speculate that enrofloxacin or toltrazuril were able to control the *T. gondii* infection in BeWo cells since these antibiotics triggered MIF production by these cells.

Additionally, a downmodulation of IL-6 in villous explants after infection by *T. gondii* and treated or not with enrofloxacin, toltrazuril, or combination of sulfadiazine plus pyrimethamine was observed in the present study. These data may represent one strategy of the parasite to subvert the immune response leading to its evasion from the protector response. This is a possible hypothesis to explain the downmodulation of cytokines in infected villi, since it can be observed in this tissue a variety of cell types, including cytotrophoblasts, syncytiotrophoblast, and macrophages. In this context, we can speculate that other mechanisms are involved in the control of parasitism in villous explants and in BeWo cells, in addition to the immune response (cytokines) triggered by the drugs. However, MIF remained upregulated when enrofloxacin was added to the villi, proving once again the importance of this cytokine in the reduction of the parasite infection.

To verify other mechanisms involved in the parasite control induced by enrofloxacin or toltrazuril, we investigated the direct effect of them on viability of the tachyzoites and structural modifications into BeWo cells. Our findings demonstrated a higher number of unviable parasites after treatment with enrofloxacin or toltrazuril in comparison with untreated parasites. Then, our data suggest that these drugs were able to alter the viability of *T. gondii* in a short treatment time, indicating that enrofloxacin and toltrazuril act not only by modulating the immune response in host cells, but they have a direct effect in *T. gondii* tachyzoites. Afterwards, structural modifications of parasites were analyzed. Treatment with enrofloxacin caused damage to tachyzoites, as “tethered” parasites, indicating difficulty completing cytokinesis, and the presence of a single membrane in some parts. Our data agree with previous study which demonstrated the effect of ciprofloxacin and its esterified compounds, a fluoroquinolone member (Martins-Duarte et al., 2015). In this study, the authors also verified structure changes in parasites treated with ciprofloxacin, as “tethered” parasites and a failure of the duple membrane (Martins-Duarte et al., 2015). We also verified “tethered” parasites in BeWo cells treated with toltrazuril, although these were at a lower frequency compared to enrofloxacin. Mitchell et al. (2004) also demonstrated a difficulty completing the normal division of tachyzoites in cells treated with ponazuril and, at the same time, verified mitochondrial damage in the tachyzoites. However, our findings did not show any changes in the mitochondria of the tachyzoites. These data may explain why enrofloxacin was better for the control of *T. gondii* proliferation in comparison to toltrazuril, since there was more damage to parasite structures treated with enrofloxacin than toltrazuril.

Taken together, our findings demonstrated that enrofloxacin and toltrazuril are able to control the *T. gondii* proliferation in BeWo cells and human villous explants, and their efficiencies are related to the upregulation of IL-6 and MIF, and a direct action on parasites, leading to damage to the tachyzoites structure. Thus, enrofloxacin and toltrazuril can be alternative strategies to prevent or treat congenital toxoplasmosis.

## Author contributions

Rd: Wrote the manuscript, performed all the experiments with RH and ME49 strains regarding to cytokine detection by ELISA, the beta-galactosidase assay for *T. gondii* intracellular proliferation and quantitative analysis (cells and villous), as well as contributed to reagent preparation. Furthermore, performed all the experiments with electron microscopy and MTT. AG, PF: Revised the manuscript and contributed to quantitative analysis. PSF: Revised the manuscript, contributed to quantitative analysis, cytokine measurement and reagent preparation. AP: Performed the experiments with ME49 strain regarding to calculate the infection index and number of tachyzoites. IM: Contributed to LDH dosage and cytokine detection. MR: Contributed to cytokine detection and experiments with ME49 strain. Md: Contributed to placenta collection, provided suggestions and valuable discussion throughout the study. JM, Nd, EF: Revised the manuscript, provided suggestions and valuable discussion throughout the study. BF: Conceived the idea of the manuscript, wrote the manuscript, performed all the cytokine detection by ELISA, the beta-galactosidase assay for *T. gondii* intracellular proliferation and quantitative analysis, as well as contributed to reagent preparation.

### Conflict of interest statement

The authors declare that the research was conducted in the absence of any commercial or financial relationships that could be construed as a potential conflict of interest.
