# Supplementary figures and images for: Enrofloxacin and Toltrazuril Are Able to Reduce Toxoplasma gondii Growth in Human BeWo Trophoblastic Cells and Villous Explants from Human Third Trimester Pregnancy
Source: Front Cell Infect Microbiol. 2017 Jul 26;7:340. doi: 10.3389/fcimb.2017.00340 (PMC5526852; doi:10.3389/fcimb.2017.00340)

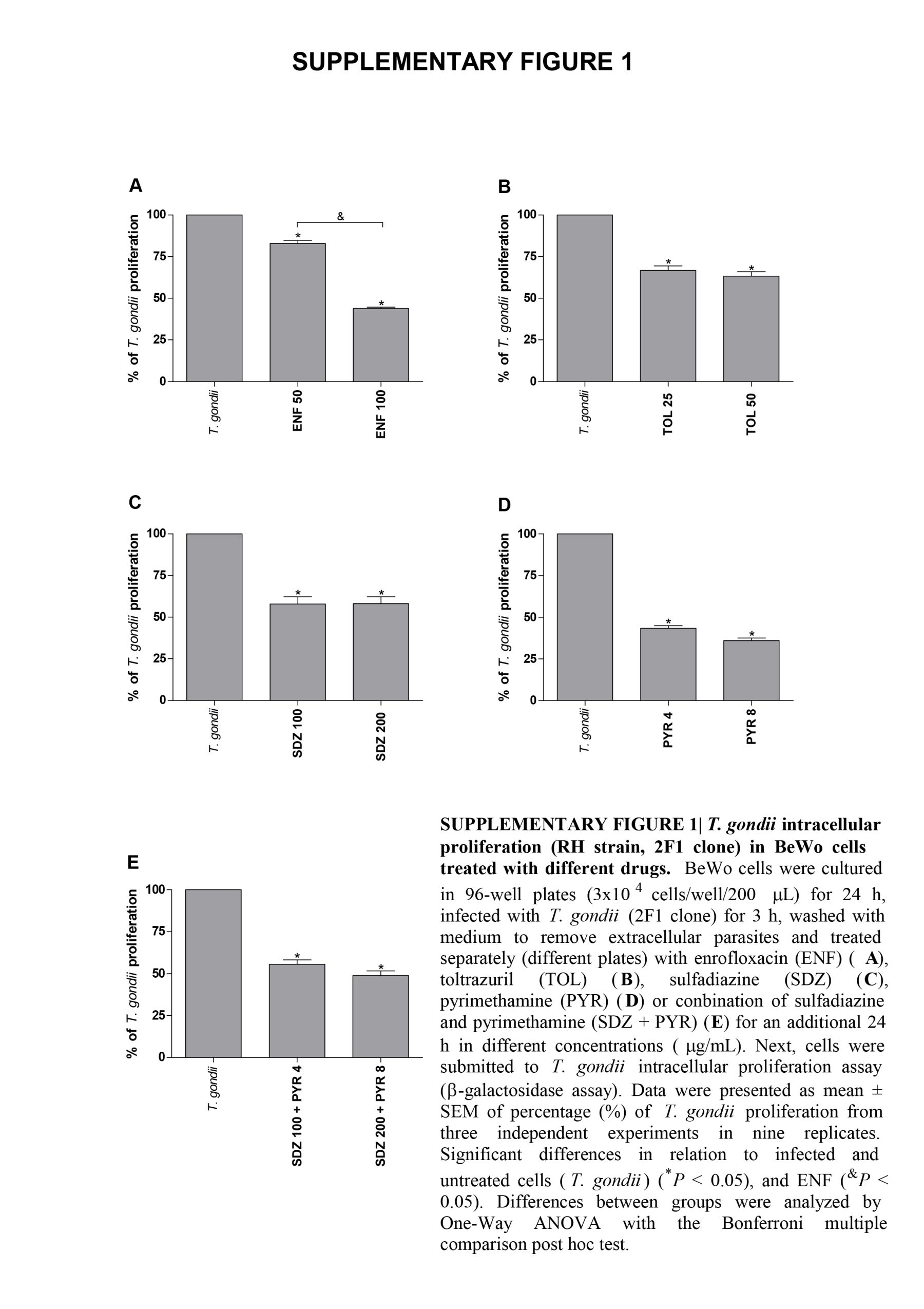

Supplement: Supplementary file 1 [file Image1.JPEG]
